# The Influence of Entrepreneurial Policy on Entrepreneurial Willingness of Students: The Mediating Effect of Entrepreneurship Education and the Regulating Effect of Entrepreneurship Capital

**DOI:** 10.3389/fpsyg.2021.592545

**Published:** 2021-07-29

**Authors:** Zhuo Zelin, Chen Caihong, Chen XianZhe, Min Xiang

**Affiliations:** ^1^Center for Timor-Leste Studies, South China Normal University, Guangzhou, China; ^2^Foreign Languages College, Minnan Normal University, Zhangzhou, China; ^3^School of Education, South China Normal University, Guangzhou, China; ^4^School of Stomatology, Wenzhou Medical University, Wenzhou, China

**Keywords:** entrepreneurship policy, entrepreneurship capital, entrepreneurship willingness, entrepreneurship education, mediating effect, regulating effect

## Abstract

With the transformation and development of the social economy of the country, innovation, and entrepreneurship have been a wide concern in all sectors of the society. In contrast, the entrepreneurial success rate of college students in China is low, and the willingness of students to start a business is generally not high. As a leading element, entrepreneurship policy plays an important role in creating an enabling social environment for entrepreneurship and promoting innovation and entrepreneurship. The influence of entrepreneurship policy on the entrepreneurial will of college students is not only reflected in the improvement of entrepreneurship environment but also in the reform and development of innovation and entrepreneurship education in colleges and universities. This study conducted a survey of fresh graduates from 1,231 colleges and universities in 31 provinces across the country to examine the path and influence the mechanism of entrepreneurship policy on entrepreneurial willingness, and the subsequent regression analysis results show that the entrepreneurship policy and entrepreneurship willingness are positively related, and entrepreneurship education, as a “bridge,” presents a partial intermediary role in the relationship. In addition, the study also found that the entrepreneurship capital of college students has a moderating effect on the path of entrepreneurship education–entrepreneurship willingness, that is, the higher the entrepreneurship capital of students is, the higher the entrepreneurship willingness will be generated after they receive entrepreneurship education.

## Introduction

In recent years, with the deepening transformation of the soft and hard environment of innovation and entrepreneurship of the government, an increasing number of college students have started to make contributions to it. As the main source of innovation and entrepreneurship, colleges and universities play an irreplaceable role in guiding innovation and entrepreneurship willingness of college students and training their related skills. In May 2015, the Central Committee of CPC and the State Council issued a document to confirm the importance of deepening the innovation and entrepreneurship education reform in institutions of higher learning for the national strategy of innovation-driven development. The next year, the Ministry of Education integrated the innovation and entrepreneurship curriculum into the curriculum system of institutions of higher learning, and colleges and universities around the country also deepened the innovation and entrepreneurship education reform. Obviously, from the support of national policies to the corresponding resource support of the university itself, which attaches great importance to innovation and entrepreneurship education, colleges and universities have ushered in unprecedented opportunities for development.

However, due to the high risk and uncertainties of innovation and entrepreneurship, university graduates are generally not willing to start a business. Most graduates choose to continue their studies or find more stable jobs (Liao et al., 2018) instead of starting a business. Among the entrepreneurial projects, there are very few that can launch and incubate, and the success of entrepreneurship is even rarer. According to the relevant survey results, the global entrepreneurial failure rate is as high as 70%, and the failure rate of Chinese college students is over 90%, and <15% of them choose to start a business again (Song et al., 2013).

The low entrepreneurial willingness of college students is the first problem encountered by Chinese policymakers in implementing innovative and entrepreneurial education policies. Relevant studies at home and abroad have shown that entrepreneurial willingness is the psychological prerequisite for entrepreneurial action and has an important predictive effect on entrepreneurial behavior (Moen, 1997; Noel, 2001). Relevant entrepreneurial policies have been published one after another recently, but for youth, if they keep low willingness to start a business, no matter how good the entrepreneurial policy is, they will not be attracted. For policymakers, it is of great significance to enhance the entrepreneurial willingness of students, stimulate enthusiasm of students, and inject vitality of students for innovation and entrepreneurship.

Entrepreneurship policy has more obvious effects on innovation and entrepreneurship education in universities. With the recent emphasis on entrepreneurship policies on innovation and entrepreneurship education of college students, the role of college innovation and entrepreneurship education in enhancing the entrepreneurial willingness of students has gradually emerged (Sun et al., 2017). It is true that innovation and entrepreneurship education, as an important external factor, has a significant influence on the entrepreneurial willingness of college students. But entrepreneurship is a highly comprehensive activity. In addition to the factor of innovation and entrepreneurship education, own entrepreneurship capital of entrepreneurs also plays an important role. Judging from the current active entrepreneurs, they all have unique and outstanding entrepreneurship capital, and those with high entrepreneurship capital often have higher entrepreneurial willingness.

This research will start with entrepreneurial policy and explore the effect of entrepreneurial policy on the entrepreneurial willingness of students through innovation and entrepreneurship education, and the effect of entrepreneurship capital in this process. The purpose of the research is to promote the role of entrepreneurship policy and to provide reference for enhancing the entrepreneurial willingness of students.

## Definitions and Literature Review

### Definitions

#### Entrepreneurship Policy

Although academia has been studying entrepreneurship policy for a long time, there is no unified definition due to different understandings of entrepreneurial activities. Some scholars make judgments on whether relevant policies are entrepreneurial policies on the basis of the relationship of policies with entrepreneurial activities (Audretsch, 2004), while some scholars make judgments based on whether the aims of policies are to promote entrepreneurship (Woolley and Rottner, 2008). Generally speaking, entrepreneurship policy refers to various incentive and supportive policies or measures made by the government to encourage an individual search for jobs and self-employment (Xu and Chen, 2015). A complete entrepreneurial policy system should include the stimulation of entrepreneurship willingness before entrepreneurial activities, and the support and protection of actions of entrepreneurs after the beginning of the entrepreneurial activities (Reynolds et al., 2005). This study combines the definitions given by several scholars to define entrepreneurship policy as such: relevant guidance, preferential, auxiliary, and protective measures promulgated and implemented by the government to develop the economy and encourage domestic or regional economic entities to carry out entrepreneurial activities.

#### Entrepreneurship Education

Entrepreneurship education refers to all means of imparting entrepreneurial knowledge. It is aimed at cultivating the entrepreneurship awareness of college students, helping them to master related knowledge and to strengthen related skills. In a narrow sense, entrepreneurship education can be defined as the education system where schools set up courses related to entrepreneurship to cultivate the entrepreneurial spirit of students and to gradually equip them with the capability to start their own business (Gorman et al., 1997). In a broad sense, entrepreneurship education refers to the educational system that cultivates the entrepreneurial skills of students, shapes their entrepreneurial values, and enhances their capability to recognize business opportunities through various ways such as entrepreneurship courses, practices, and competitions (Brown, 2000). Some scholars believe that entrepreneurship education should not only impart entrepreneurial strategies and thinking, which are conventional contents, but also should cultivate entrepreneurial spirit and attitudes of students (Riahi, 2010). Based on this, the study defines entrepreneurship education as such: an education system that cultivates entrepreneurial skills, thinking, spirit, and attitude of students through various ways such as entrepreneurship courses, practices, and competitions.

#### Entrepreneurship Willingness

Entrepreneurship willingness originates from psychology and is the integration of entrepreneurial and psychological research. Dating back to 1988, Bird defined entrepreneurship willingness as a mental state that can guide entrepreneurs to strive for their goals and can influence entrepreneurial behavior (Brid, 1988). Some other researchers believe that entrepreneurship willingness is a prerequisite for implementing entrepreneurial behavior and a subjective attitude of entrepreneurs on whether to engage in entrepreneurial activities (Bagozzi and Kimmel, 1995). This study draws on the viewpoint of Qian to define entrepreneurship willingness as a subjective attitude and expectation of potential entrepreneurs of whether to engage in entrepreneurial activities, a simple predictor of entrepreneurial behavior, and a significant core for understanding the entrepreneurial process (Qian, 2007).

#### Entrepreneurship Capital

Academic research on entrepreneurship capital at present mainly focuses on the personal capital and family capital of students. Pierre first proposed the concept of family capital when studying the theory of social capital. He believes that family capital is composed of family economic capital, social capital, and cultural capital (Pierre, 1997). Patrick thinks that personal capital is the sum of different kinds of capital owned by entrepreneurs before starting a business, including economic capital, human capital, and social capital. Since college students have not yet established their own complete economic and social capital, their entrepreneurship capital is more reflected in their human capital. Therefore, this study defines the entrepreneurship capital of college students as their inborn human and family capital brought by their family background during entrepreneurship process.

## Literature Review and Research Hypothesis

Since 2015, the CPC Central Committee and the State Council have issued a series of preferential policies for innovation and entrepreneurship, which have optimized the investment environment, standardized entrepreneurial platforms, provided in-depth entrepreneurial services, providing good opportunities for university students to start a business. Yang of the National Chengchi University conducted a meta-analysis to study the relationships between entrepreneurship policy, entrepreneurship education, and entrepreneurship willingness for the past decade and discovered that the positive correlation effects between entrepreneurship policy, education, and entrepreneurship willingness have reached the medium level (Yang, 2016). The results of research done by Xie et al. on entrepreneurship willingness of college students in Henan Province show that policy support has a significant positive influence on the entrepreneurship willingness of university students (Xie et al., 2018). The possible reason why entrepreneurship policy has a positive influence on entrepreneurship willingness is that the stronger the policy support, the fewer the entrepreneurial risks and uncertainties faced by college students, and the lower the transaction costs of entrepreneurship. It will boost the enthusiasm of college students for starting a business and increase their entrepreneurship willingness. According to this, the study proposes the first research hypothesis: Entrepreneurship policy has a positive influence on entrepreneurship willingness of college students.

On the other hand, entrepreneurship policy also plays a significant supporting role in innovation and entrepreneurship education in colleges and universities. Entrepreneurship policy has always attached great importance to the reform and support of innovation and entrepreneurship education. With the release of reform policies on innovation and entrepreneurship education in recent years, innovation and entrepreneurship education have also developed rapidly. In addition to conventional entrepreneurship courses, entrepreneurial competitions are in full swing among universities and colleges across the country. Universities have set up business incubators to support innovative and entrepreneurial activities of students. Zhu Guanghua et al. believe that currently, entrepreneurship policy has a positive influence on the support of innovation and entrepreneurship education (Zhu et al., 2015). By the promotion of innovation and entrepreneurship education, entrepreneurship policy enables students to receive more comprehensive and high-quality innovation and entrepreneurship education, which helps to enhance the entrepreneurial capability of students indirectly and thus strengthen their entrepreneurship willingness. According to this, the second research hypothesis is proposed: Entrepreneurship policy has a positive influence on entrepreneurship education.

Entrepreneurship education in colleges and universities is closely related to the entrepreneurship willingness of students and is also one of the factors that can directly affect it. The influence of entrepreneurship education on the entrepreneurship willingness of students is mainly reflected in two aspects. On the one hand, entrepreneurship education can improve the entrepreneurial skills and abilities of entrepreneurs to facilitate the development of entrepreneurial activities. It can also help people who receive such education choose suitable business types, get equipped with entrepreneurial knowledge, and thereby strengthen their own entrepreneurship willingness. On the other hand, entrepreneurship education influences the entrepreneurship willingness of students by changing the attitudes of people to entrepreneurship, subjective norms, and perceived behavior (Fiet, 2001). With the development of entrepreneurship education in recent years, studies on the relationship between entrepreneurship education and entrepreneurship willingness have emerged. Most studies have verified the positive effect of entrepreneurship education on entrepreneurship willingness (Li et al., 2018; Zhang et al., 2018). According to this, the study proposes the third research hypothesis: Entrepreneurship education has a positive influence on entrepreneurship willingness.

On the basis of the three hypotheses discussed above, it is easy to find that the influence of entrepreneurship policy on entrepreneurship willingness of students can also be reflected in its relationship with entrepreneurship education. By improving entrepreneurship education, entrepreneurship policy can also indirectly strengthen entrepreneurship willingness. Therefore, the study proposes the fourth hypothesis: Entrepreneurship education plays an intermediary role in the relationship between entrepreneurship policy and entrepreneurship willingness.

It should be recognized that entrepreneurship willingness, as a complex psychological phenomenon, is not only affected by entrepreneurship education but also influenced by the important internal factor of entrepreneurship capital of students. There is no doubt that students with different entrepreneurship capital have different levels of acceptability for entrepreneurship education. In China, the family environment formed with parents as the core plays an important role in the growth and development of children, especially in terms of entrepreneurship. Hoffmann mentions that parents with business or entrepreneurial background make good examples for their children, pass down part of their economic foundation and personal connection, and create many advantages for their children such as helping them to be mentally prepared and offering a summary of indirect experience (Hoffmann et al., 2015). The studies of different scholars on family capital of college students all show the positive influence of family capital on the entrepreneurship willingness of students (Huang, 2015; Xie, 2018). By allowing their children to meet more role models, acquire more knowledge, and accumulate corresponding capital, family capital has a subtle influence on the entrepreneurship willingness of students. In addition to the studies on family capital discussed above, personal capital, as a manifestation of personal entrepreneurship capital, is also vital in guiding the entrepreneurship willingness of college students. Wang and Li (2012) believe that the personal capital of college students is mainly reflected in the four aspects of entrepreneurial personality, attitude, capability, and knowledge. On the basis of this, they propose and verify the intermediary role of entrepreneurial endowment on entrepreneurship education and willingness and the regulatory role of personal background. In general, the effect of personal capital on entrepreneurship willingness is mainly reflected in the improvement of entrepreneurial capability and resources of students, which in turn enhances their entrepreneurship willingness. According to this, the fifth hypothesis is proposed: Entrepreneurship capital of students regulates the positive correlation between entrepreneurship education and entrepreneurship willingness.

**Figure 1:**
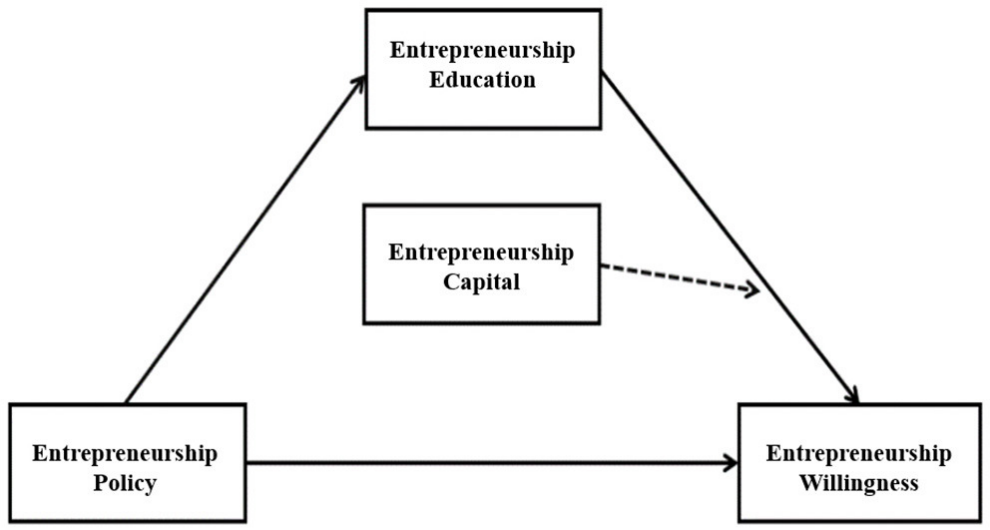
Moderated mediation model of entrepreneurship education, policy, and willingness.

According to the correlation between hypothesis 4 and hypothesis 5, we can develop a moderated mediation model with four variables: Entrepreneurship capital regulates the intermediary role of entrepreneurship education on entrepreneurship policy and entrepreneurship willingness (shown in Figure 1). The interaction process is as follows: Entrepreneurship education, as an intermediary, conveys the influence of entrepreneurship policy on entrepreneurship willingness, but the process will also be affected by entrepreneurship capital. For students with higher entrepreneurship capital, they have a stronger sense of recognition and a higher level of acceptability for entrepreneurship education, which makes it easier for them to transform their huge entrepreneurship capital into the stimulus to starting their own business. Therefore, they tend to have stronger entrepreneurship willingness. Conversely, students with low entrepreneurship capital find it more difficult to take full advantage of entrepreneurship education. Since their family and personal backgrounds drive them to seek a stable workplace, their willingness to start a business is relatively low.

## Research Objects and Measurement Tools

### Research Objects

From September 15, 2018, to January 18, 2019, the research group conducted a survey by using Wenjuanxing, a platform providing functions equivalent to Amazon Mechanical Turk, among 1,231 college students and graduates from 31 provinces (autonomous regions or direct-controlled municipalities) who have received innovation and entrepreneurship education. Results were restricted by checking IP addresses, and 187,914 questionnaires were collected, 17,150 of which were invalid because of too short answer time or invalid school name, and 170,764 of which were valid, accounting for 90.87%.

This study mainly assesses the entrepreneurial willingness and entrepreneurship capital of the students. It is found that the entrepreneurial willingness of college students in the first 2 years of college has not been finalized and may be affected by personal growth experience and entrepreneurship education received at university, so their entrepreneurial willingness is still very likely to change. Therefore, this study only examines the data of fresh graduates and junior college graduates. After using SPSS 22.0 to screen out, a total of 29,255 results were obtained.

**Figure 2:**
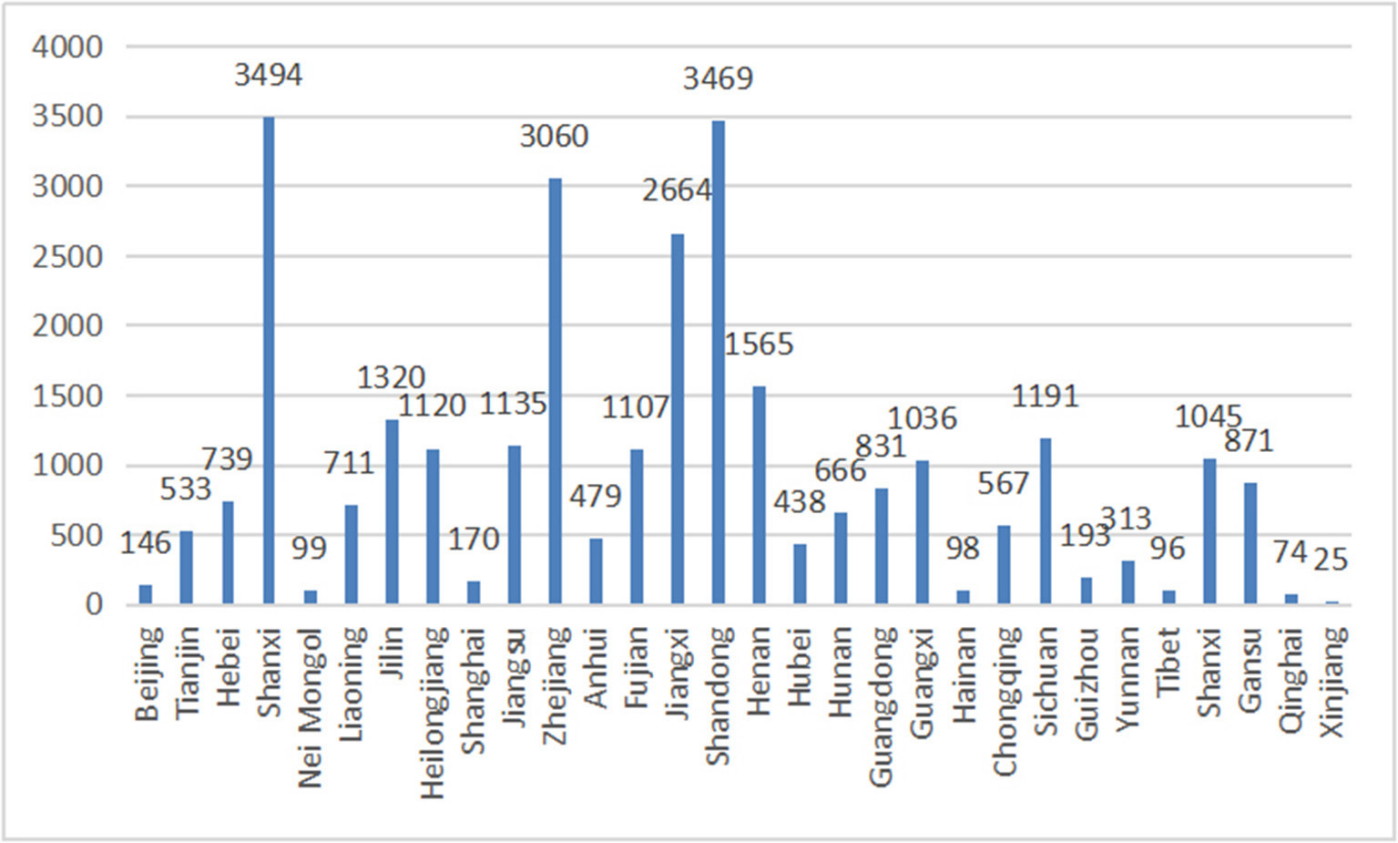
Distribution map of provinces where the sample schools are located.

Among them, 9,986 are male, accounting for 34.1%, and 19,269 are female, accounting for 65.9%; 9,163 are only children, accounting for 31.3%, and 20,092 are not only children, accounting for 68.7%; 6,007 have entrepreneurial practice experience, accounting for 20.5%; 23,248 have no entrepreneurial practice experience, accounting for 79.5%; 8,373 hold urban household registration, accounting for 28.6%; and 20,883 hold urban household registration, accounting for 71.4%; 1,345 are from “Double First-Class” universities, accounting for 4.6%; 11,131 are from common universities, accounting for 38.0%; 12,411 are from higher vocational school or colleges, accounting for 42.4%; 1,467 are from independent colleges, accounting for 5.0%; 70 are major in philosophy, accounting for 0.2%; 3,911 are major in economics, accounting for 13.4%; 235 are major in law, accounting for 0.8%; 3,048 are major in education, accounting for 10.4%; 1,504 are major in literature, accounting for 5.1%; 187 are major in history, accounting for 0.6%; 2,741 are major in science, accounting for 9.4%; 5,646 are major in engineering, accounting for 19.3%; 780 are major in agriculture, accounting for 2.7%; 3,843 are major in medical science, accounting for 13.1%; 35 are major in military science, accounting for 0.1%; 5,415 are major in management, accounting for 18.5%; 1,840 are major in art, accounting for 6.3%. In addition, the distribution of the provinces where the research objects are located is shown in Figure 2, including 30 provinces across the country. For Ningxia Autonomous Region, only freshmen and sophomores have filled in the questionnaire; therefore, the data of graduates are missing.

### Measurement

#### Entrepreneurship Policy Scale

Stevenson's Entrepreneurship Promotion Policy Scale (Stevenson, 2005) was referenced in the research. The scale includes financial and tax support, training, and entrepreneurial willingness to stimulate. It has a total of 11 questions, all of which use the method of the Likert-type scale (from “1” completely disagree to “5” strongly agree). Among them, first to third questions are related to financial and tax support, their coefficient of alpha is 0.925; fourth to sixth questions are related to training, and their coefficient of alpha is 0.899; 7th to 11th questions are related to entrepreneurial willingness to stimulate, and their coefficient of alpha is 0.952. The coefficient of alpha of the whole scale is 0.969.

#### Entrepreneurship Education Scale

Entrepreneurship education is generally measured by the satisfaction of students with school innovation and entrepreneurship education. In this study, Huang and Huang (2019) Entrepreneurship Education Evaluation Scale was referenced to measure a total of 25 topics, with the method of the Likert-type scale (from “1” completely disagree to “5” strongly agree). Among them, first to sixth questions are related to entrepreneurial courses, and their coefficient of alpha is 0.930; seventh to 14th questions are related to entrepreneurial competition, and their coefficient of alpha is 0.945; 15th to 20th questions are related to entrepreneurial practice, and their coefficient of alpha is 0.939; 21st to 25th questions are related to teacher resources, and their coefficient of alpha is 0.945. The coefficient of alpha of the whole scale is 0.970.

#### Entrepreneurship Willingness Scale

Per Davidsson's Entrepreneurial Willingness Scale was referenced in the research. In his empirical studies, this scale passed the reliability and validity test and the results reached a high level (Davidsson, 2003). There are four questions in the scale, with the method of the Likert-type scale (from “1” completely disagree to “5” strongly agree). The coefficient of alpha of the whole scale is 0.853.

#### Entrepreneurship Capital Scale

Entrepreneurship capital consists of personal capital and family capital. This study includes five questions about personal capital: entrepreneurial practice experience, innovation and entrepreneurship research team, the type of school attended, university academic performance, and entrepreneurial resources mastered. The first two questions used the two-point scale, while the other three used the two-point scale.

### Data Processing

SPSS22.0 was used to conduct the tests of common method bias, descriptive statistics, and correlation analysis of each variable, regression analysis, and so on.

## Data Analysis and Hypothesis Testing

### Common Method Bias Test

The use of self-report data may lead to common method bias; therefore, first, Harman's single factor test was used and it was found that there are eight factors with eigenvalues >1 without rotation, and the percentage of the first factor variation explained is 22.1%, which is <40% of the judgment standard recommended by Harris (1996), indicating that the problem of common method bias in this study is not obvious.

### Related Analysis

After descriptive statistics and correlation analysis of variables such as control variables, entrepreneurial willingness, entrepreneurship capital, entrepreneurial education, and entrepreneurial policy, the results of the mean, SD, and correlation coefficient of each variable are shown in Table 1. Among them, entrepreneurial willingness and entrepreneurship capital, entrepreneurial policy, and entrepreneurial education are positively correlated.

**Table 1:** Correlation matrix between entrepreneurial willingness and entrepreneurship capital, entrepreneurial policy, and entrepreneurial education satisfaction.

**Variables**	***M***	***SD***	**1**	**2**	**3**	**4**	**5**	**6**
1. Gender	1.66	0.474	1					
2. Only-child	1.69	0.464	0.161^***^	1				
3. Entrepreneurial willingness	23.36	12.47	−0.134^***^	−0.054^***^	1			
4. Entrepreneurship capital	11.90	1.87	−0.074^***^	0.032^***^	0.476^***^	1		
5. Entrepreneurial policy	40.96	8.289	−0.032^***^	−0.025^***^	0.367^***^	0.305^***^	1	
6. Entrepreneurial education	85.31	16.70	−0.056^***^	−0.042^***^	0.318^**^	0.213^***^	0.802^***^	1

* *p < 0.1*,

** *p < 0.05*,

*** *p < 0.01*.

#### Regulated Intermediary Model Test

According to the views of Wen and Ye (2014), testing the regulated intermediary model requires the estimation of the parameters of the three regression equations (Wen and Ye, 2014). Equation 1 estimates the moderating effect of the adjustment variable on the relationship between the independent variable and the dependent variable. Equation 2 estimates the moderating effect of moderating variables on the relationship between independent variables and intermediary variables. Equation 3 estimates the adjustment effect of the adjustment variable on the relationship between the intermediary variable and the dependent variable, and the adjustment effect of the independent variable on the residual effect of the dependent variable.

**Figure 3:**
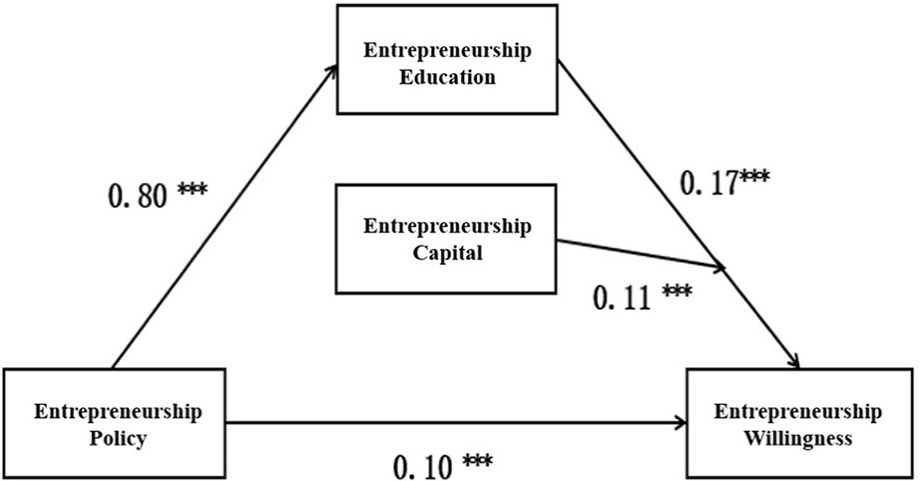
Regulated intermediary model. ***p < 0.01.

In this study, SPSS 22.0 was used to statistically analyze each variable and perform regression analysis on entrepreneurial willingness to obtain a regression model of entrepreneurial willingness. As a result, in each model, the first step is to add control variables (gender and only-child as dummy variables) and then add predictors into the model. All variables included in the cross-terms are centered before regression. The results obtained are as follows:

(1) The benchmark model includes two control variables related to independent variables: gender and only-child; the benchmark model is significant, *F*_(4, 29, 252)_ = 282.59, *p* < 0.001, *R*^2^ = 0.019;
(2) Equation 1 is overall significant, *F*_(4, 29, 251)_ = 4160.77, *p* < 0.001, *R*^2^ = 0.31, Δ *R*^2^ = 0.29; the regression coefficient is significant, β = 0.24, *t* = 48.47, *p* < 0.001. It shows that entrepreneurial policies can positively affect entrepreneurial willingness, which can verify the first hypothesis.
(3) Equation 2 is overall significant, *F*_(4, 29, 251)_ = 22593.46, *t* = 28.12, *p* < 0.001, *R*^2^ = 0.70, Δ *R*^2^ = 0.69; the regression coefficient is significant, β = 0.240, *t* = 245.71, *p* < 0.001, indicating that entrepreneurship policies can positively predict the entrepreneurship education, which can verify the second hypothesis.
(4) Equation 3 is overall significant, *F*_(4, 29, 251)_ = 2619.95, *p* < 0.001, *R*^2^ = 0.32, Δ *R*^2^ = 0.30; regression coefficient is significant, β = 0.170, *t* = 18.93, *p* < 0.001, indicating entrepreneurship education can positively predict the willingness to start a business, which can verify the third hypothesis. Combining the first two hypotheses, we can see the intermediary variable of entrepreneurship education in the relationship between entrepreneurship policy and entrepreneurial willingness; therefore, the fourth hypothesis can be verified, and since entrepreneurial policies still have a significant influence on entrepreneurial willingness, β = 0.10, *t* = 10.99, and *p* < 0.001, so entrepreneurship education plays a partial intermediary role. In addition, the interaction between entrepreneurship education and entrepreneurship capital is also significant, β = 0.11, *t* = 12.13, *p* < 0.001, which shows that entrepreneurship capital regulates the path from entrepreneurial education to entrepreneurial willingness, which can verify the fifth hypothesis, and the regulated intermediary model test is shown in Table 2, the regulated intermediary model is shown in Figure 3.

**Table 2:** Regulated intermediary model test.

	**Equation 1 (Dependent variable: entrepreneurial willingness)**		**Equation 2 (Dependent variable: entrepreneurial education)**		**Equation 3 (Dependent variable: entrepreneurial willingness)**	
	**β**	***t***	**β**	***t***	**β**	***t***
Gender^a^	−0.08	−17.36^***^	−0.01	−5.13^***^	−0.08	−5.13^***^
Only-child^b^	−0.04	−8.89^***^	−0.02	−6.61^***^	−0.04	−8.05^***^
Entrepreneurial policy	0.24	48.47^***^	0.80	245.71^***^	0.10	10.99^***^
Entrepreneurship capital	0.40	80.35^***^	0.11	33.91^***^	0.38	75.40^***^
Entrepreneurial policy ^*^ Entrepreneurship capital	0.14	28.12^***^	0.05	14.93^***^	0.04	4.20^***^
Entrepreneurial education					0.17	18.93^***^
Entrepreneurial education ^*^ Entrepreneurship capital					0.11	12.13^***^
*R^2^* (variation)	0.29		0.69		0.30	
*F*	4160.77^***^		22593.46^***^		2619.95^***^	

* *p < 0.1*,

** *p < 0.05*,

*** *p < 0.01*.

**Figure 4:**
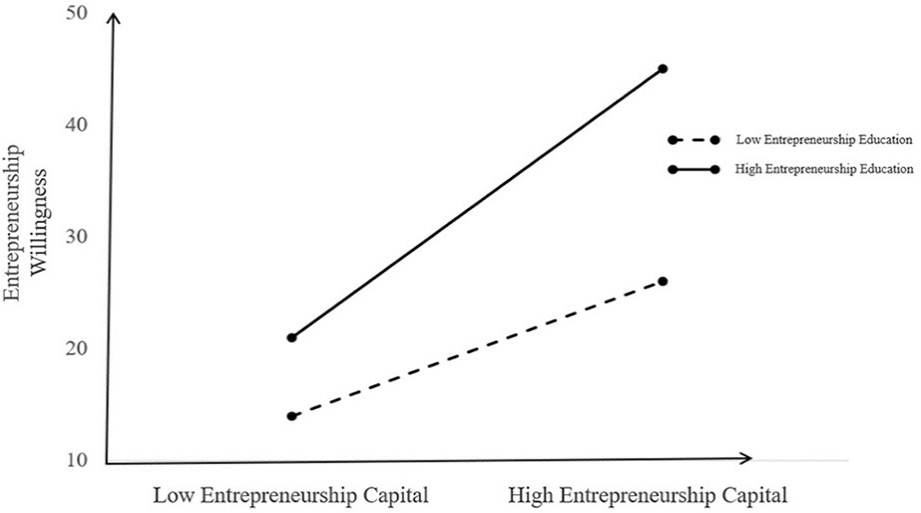
The regulatory role of entrepreneurship capital in entrepreneurship education on entrepreneurial willingness.

In order to more clearly reveal the regulatory role of entrepreneurship capital in the influence of entrepreneurship education on entrepreneurial willingness, entrepreneurship capital is divided into high and low groups according to a positive and negative standard difference, and a simple slope test is conducted to examine the influence of entrepreneurship education on entrepreneurial willingness at different levels of entrepreneurship capital. A simple-effect analysis chart is shown in Figure 4. The test results show that in the low entrepreneurship capital group, entrepreneurship education can positively predict entrepreneurial willingness, that is, as perceived entrepreneurial education increases, entrepreneurial willingness changes significantly, β = 0.28, *t* = 24.89, *p* < 0.001; entrepreneurship willingness increases by only 0.28 standard deviations for each additional standard deviation of entrepreneurship education; in the high entrepreneurship capital group, entrepreneurial education can also positively predict entrepreneurial willingness, that is, as perceived entrepreneurial education increases, entrepreneurial willingness changes significantly, β = 0.41, *t* = 34.26, *p* < 0.001; entrepreneurship willingness increases by 0.41 standard deviations for each additional standard deviation of entrepreneurship education. Therefore, as entrepreneurial education of university students improves, those with high entrepreneurship capital will increase their entrepreneurial willingness faster than those with low entrepreneurship capital.

Therefore, the entrepreneurial policy is regulated by entrepreneurship capital through the influence of entrepreneurship education on entrepreneurial willingness. For individuals in the low entrepreneurship capital group, the improvement in entrepreneurial education is conducive to enhancing entrepreneurial willingness. For individuals with high entrepreneurship capital, entrepreneurial education has a stronger role in promoting entrepreneurship. Combined with the test result of the intermediary role of entrepreneurship education on the relationship between entrepreneurship capital and entrepreneurial willingness, Bootstrap in SPSS22.0 was set to 1,000 times. The indirect effect of entrepreneurship capital on entrepreneurial willingness through entrepreneurial policies is significant, with a value of 0.27 [*p* < 0.05, bias-corrected CI = [0.08, 0.34]], and the relationship between entrepreneurship education and entrepreneurial willingness is positively regulated by entrepreneurship capital, which is manifested as a regulated intermediary mode.

## Conclusion and Discussion

### Entrepreneurship Education Plays an Intermediary Role in the Relationship Between Entrepreneurship Policy and Entrepreneurial Willingness

The promotion of entrepreneurial willingness has always been a study subject of much concern. Improving the entrepreneurial willingness of students is of great significance to the current Chinese society. Its significance not only lies in easing employment pressure but also in conforming to the trend of the new era and encourages students to demonstrate the spirit and entrepreneurial ability, thus accomplishing a new historic mission. With the encouragement of the government, entrepreneurship policies have been continuously introduced, providing better conditions for entrepreneurs and potential entrepreneurs. In particular, the continuous introduction of preferential policies for young entrepreneurs has greatly stimulated the entrepreneurship vitality of Chinese youth, and an increasing number of entrepreneurs continue to emerge with their own projects across the country. At present, the evaluation research on the effect of entrepreneurial policies springs up. However, there is a lack of research on how entrepreneurial policies affect entrepreneurial willingness (Yang, 2016). Based on the investigation of 1,231 colleges and universities in 31 provinces across the country, this study attempts to find a possible path of influence of entrepreneurial policy on entrepreneurial willingness through empirical data analysis and reveals the partial intermediary role of entrepreneurial education in entrepreneurial policy and entrepreneurial education. That is to say, in the process of influencing entrepreneurial willingness, entrepreneurial policies, on the one hand, directly improve the entrepreneurial enthusiasm of students by publicity campaign and other promotion activities, thereby enhancing entrepreneurial willingness of students; on the other hand, entrepreneurial policies also have an influence on the innovation and entrepreneurship education in colleges and universities, through policy reform and policy support of innovation and entrepreneurship education, enables innovation and entrepreneurship education to enhance the entrepreneurial ability and entrepreneurial enthusiasm of students, which in turn affects the entrepreneurial willingness of students. In this context, entrepreneurship education serves as a bridge. Colleges and universities should carry out entrepreneurship policies to the letter because students will have higher entrepreneurial willingness if their colleges or universities have effective entrepreneurship education.

### Entrepreneurship Capital Regulates the Second Half of the Path of Intermediary Role Entrepreneurship Education Plays Between Entrepreneurship Policy and Entrepreneurial Willingness

The complex nature of entrepreneurial willingness determines that it is affected by internal and external factors. In addition to external factors, the influence of individual internal factors on entrepreneurial willingness should also be considered (Ye and Fang, 2017). Based on the intermediary role of entrepreneurship education, this study introduces own entrepreneurship capital of students and attempts to construct a regulated intermediary model. It is found that entrepreneurship capital has a significant regulatory effect on the path of entrepreneurship education acting on entrepreneurial willingness. The moderating effect of entrepreneurship capital on the relationship between entrepreneurship education and entrepreneurial willingness is mainly manifested in the fact that students with high entrepreneurship capital are more likely to generate entrepreneurial willingness than students with low one under the premise of students receiving the same entrepreneurial education. With the same investment in entrepreneurship education, students with high entrepreneurship capital can better transform it into a motivation to promote entrepreneurial willingness. Entrepreneurship capital is closely related to family capital and personal capital of students. From the perspective of family capital, students with high entrepreneurship capital have a higher socioeconomic status and more entrepreneurial resources and entrepreneurial models they can access. Based on the accumulation of such entrepreneurial resources, students can practice skills better when receiving entrepreneurial education. They can better combine the theoretical knowledge of entrepreneurship education with life experience and have the advantage of transforming the entrepreneurship capital of family to help entrepreneurship. Therefore, the entrepreneurial willingness of students with high entrepreneurship capital is more likely to be stimulated by entrepreneurship education.

It is necessary to point out that students with higher entrepreneurship capital also have stronger personal abilities, and the entrepreneurial experience and entrepreneurial capabilities they possess can better assist them in receiving entrepreneurial education, thereby forming a virtuous circle. Entrepreneurship education has increased their entrepreneurship competence, and the increment of entrepreneurial ability, in turn, enhances their understanding and acceptance of entrepreneurship education, thereby improving the entrepreneurial willingness of students. On the contrary, because students with low entrepreneurship capital have low socioeconomic status, which cannot bring good family capital to them, and may even have a negative effect. Families of some students have difficulties in finance, which results in their tendency in choosing stable jobs to share the financial pressure of the family. Such students have a relatively indifferent attitude toward entrepreneurship courses and a relatively low acceptance level. It is naturally difficult to enhance their entrepreneurial willingness through entrepreneurship education.

### Countermeasures and Suggestions

#### Strengthen the Support of Entrepreneurship Education Policy and Implement the Entrepreneurship Education Policy

With the recent emphasis of the country on innovation and entrepreneurship, the introduction of entrepreneurship policies has become increasingly frequent. The guidance and support of the government are very important for the innovation and entrepreneurship of college students. It is necessary to continue to increase support for students to start a business, optimize the allocation of entrepreneurial resources, timely grasp the entrepreneurial needs of students, and provide necessary entrepreneurial funding support. Entrepreneurship policies that can bring benign effects should be ensured to be put in place. And for those with problems, improvement should be made. At present, there is a lack of unified publishing of resources and legal documents to consolidate entrepreneurial policies, resulting in the fact that the current entrepreneurial policies are not integrated as a whole and lack a coupling effect. In terms of implementation, due to the above two reasons, some policies have not been implemented completely and the effect has been greatly reduced. To play the role of entrepreneurship policy effectively, it is required that relevant departments coordinate each policy participant, clarify relevant responsibilities, optimize the policy system, and ensure that the policy can be implemented effectively. In addition, from the level of the law, relevant legislation work should be made to provide a guarantee mechanism for the development of innovation and entrepreneurship, which act as a shot in the arm for all entrepreneurs. At the same time, improvement of entrepreneurship education policies and reforms of entrepreneurship education in colleges and universities should be made. Construction of innovation and entrepreneurship service system should be strengthened, and collaborative personnel training mode should be improved to stimulate the innovation and entrepreneurship of students.

#### Promote Reforms and Development of Entrepreneurship Education and Give Full Play to the Education Function of Entrepreneurship Education

Entrepreneurship education, as an intermediary factor between entrepreneurship policy and entrepreneurial willingness, has a linking role as a “bridge.” Therefore, entrepreneurship education should play the role of linking government policies with the needs of students. It should be conducted according to government policies, implemented the requirements of entrepreneurship education policies completely to effectively create “golden courses” for students in first-class entrepreneurship education; excellent entrepreneurial faculty should be introduced to creating good entrepreneurship atmosphere and efficient communicating platforms of entrepreneurial resources. The entrepreneurial policy bonus should be released through various channels to bring effective entrepreneurial preferential treatment and support to students. At the same time, the educational function of entrepreneurship education should be made good use of in order to reverse the utilitarian tendency of pursuit of success rate of startups of students and cultivate the entrepreneurial awareness, innovative spirit, and innovative entrepreneurial ability of college students in an all-round way. It is necessary to point out that to give full play to the educational function of entrepreneurship education, entrepreneurial teachers (mentors) are required to keep in mind the educational mission of innovative entrepreneurship in the education process, focus on stimulating innovative and entrepreneurial awareness of students, and carefully analyze the innovation factors of each course, and tap the entrepreneurial potential of students. And in the process of education, students will be trained about the quality of will, professional ethics, social responsibility, and core socialist values for innovation and entrepreneurship, so that students can grow into true entrepreneurs with socialist core values.

#### Pay Attention to the Entrepreneurial Plight of Students With Low Entrepreneurship Capital and Enhance the Overall Entrepreneurial Willingness of University Students

From the conclusions of this study, it is found that entrepreneurship capital has an influence on the path of entrepreneurship education acting on entrepreneurial willingness. The regulatory role of entrepreneurship capital makes it easier for students with high entrepreneurship capital to have the entrepreneurial willingness, while students with low entrepreneurship capital have a lower entrepreneurial willingness. Based on this situation, the government and universities should increase their focus on students with low entrepreneurship capital, increase their support of start-up capital and loan concessions for such entrepreneurs, and increase the entrepreneurial willingness of students by easing their family economic pressure. At the same time, the entrepreneurial training base of school is used to provide students with opportunities for entrepreneurial practice, and entrepreneurial skills and entrepreneurial qualities of students with low entrepreneurship capital can be trained through entrepreneurial practice and competitions to further enhance their entrepreneurial willingness. In addition, different entrepreneurship education programs should be conducted aiming at students with different entrepreneurship capital. For course design, students can choose appropriate entrepreneurship courses according to their interest types of business. For students with low entrepreneurship capital, they should have access to both entrepreneurship theory and practice, accumulate entrepreneurship capital, and enhance entrepreneurial skills to increase their entrepreneurial willingness. For students with high entrepreneurship capital, they should be provided with platforms that are opportunities to get in touch with resources and, accelerate the formation of entrepreneurial projects so as to increase their entrepreneurial willingness.

## Data Availability Statement

The raw data supporting the conclusions of this article will be made available by the authors, without undue reservation.

## Ethics Statement

The studies involving human participants were reviewed and approved by Ethics Committee of South China Normal University. Written informed consent to participate in this study was provided by the participants.

## Author Contributions

In the process of data search, paper writing, and revision of this paper, MX has made great contributions. All authors contributed to the article and approved the submitted version.

## Conflict of Interest

The authors declare that the research was conducted in the absence of any commercial or financial relationships that could be construed as a potential conflict of interest.

## Publisher's Note

All claims expressed in this article are solely those of the authors and do not necessarily represent those of their affiliated organizations, or those of the publisher, the editors and the reviewers. Any product that may be evaluated in this article, or claim that may be made by its manufacturer, is not guaranteed or endorsed by the publisher.
